# Kinetics of Photodegradation and Durability of Inkjet Prints: A Comparative Study of Aqueous Solutions and Printed Substrates

**DOI:** 10.3390/molecules30040968

**Published:** 2025-02-19

**Authors:** Barbara Blaznik, Franci Kovač, Sabina Bračko

**Affiliations:** 1 Faculty of Natural Sciences and Engineering, University of Ljubljana, Snežniška 5, 1000 Ljubljana, Slovenia; barbara.blaznik@ntf.uni-lj.si; 2 Faculty of Chemistry and Chemical Technology, University of Ljubljana, Večna pot 113, 1000 Ljubljana, Slovenia; franci.kovac@gmail.com

**Keywords:** photodegradation, daylight, inkjet, aqueous solution inks, chromatography, prints

## Abstract

The durability of the materials is often limited as they fade under the influence of external factors, particularly light. The present research aimed to study the photodegradation of commercial inkjet inks in an aqueous solution. The results were compared with their stability on prints in order to establish the connection between the kinetics of photodegradation of dye in the solution and the durability of the final print. Thin-layer chromatography (TLC), chromatography with a mass selective detector (GC/MS), and spectrophotometric measurements were used to study the effect of light, including near UV. The results clearly show that the catalytic effect between different dyes cannot be avoided, as the inks for inkjet printing are usually a mixture of different colorants. A comparison of the results of photodegradation of the dye in solution and on the final prints does not show a direct connection due to the different influences of external factors. Consequently, it was established that it is not possible to predict the photodegradation of prints solely based on a single dye’s analysis in solution. The paper as a substrate must be included in the analysis, as it significantly influences the photodegradation of the print.

## 1. Introduction

The prints produced by an inkjet printer are determined by the color range, sharpness of an image, and print durability, which requires ink to bind to the surface and to be absorbed quickly into the substrate. Therefore, the quality of prints depends mainly on the properties of the ink and paper and their interactions [1,2,3,4,5,6]. Inks for inkjet printing can be classified according to the dye- or pigment-based colorants. Generally, dyes are less stable than pigments but are soluble in different solvents [3].

Many interrelated external and internal factors influence the fastness of prints, and it is not easy to assess the impact and importance of only one of them. Photodegradation can be accelerated by heat, moisture, and oxygen [3,7,8,9,10]. Moreover, the chemical structure of the printing material and ink, the penetration of ink into the substrate, the chemical properties of the environment in which the ink is located, and many other components can also influence the photodegradation of prints [1,11,12,13,14]. Light combined with other external factors has been shown to change the material significantly; particularly, light with shorter wavelengths is very destructive [11,15]. We also encounter catalytic fading of prints in the inkjet printing process based on subtractive mixing of primary colors [3,16,17,18]. This means that energy is transferred from one molecule to its surroundings; therefore, the absorbed energy in the dye can be transferred to another dye [16,17]. On the other hand, dye mixtures have been developed to give higher photostability than single colorants alone [19]. Although damage to paper due to light exposure is considered a minor factor, it should not be neglected when archiving printed material [8,20,21]. Photodegradation of paper causes a change in the acidity of the paper, triggering chemical reactions that affect its optical and mechanical properties [8,21,22,23], which occur mainly due to the concentration of additives, such as optical brighteners (OBAs), in the paper [22,24,25].

Despite substantial research over the last years, we still cannot precisely explain the role of key factors that influence the properties of materials and, consequently, the fastness of prints. Our research aimed to study commercial inks in aqueous solution compared to prints to investigate the impact of light as the most destructive external factor. Therefore, we focused on two commercial inks: cyan and magenta. Cyan serves as an example of the more lightfast process inks, while magenta is generally considered the most critical ink regarding lightfastness on the print [26,27,28]. For the analysis of the inks, thin-layer chromatography (TLC) and gas chromatography with a mass selective detector (GC/MS) methods were used. For the exposure of samples, different wavelength ranges (λ > 300 and λ > 320) of light were used. After exposure, the amount of ink and the half-lives in the solution and on the prints were determined using spectrophotometric measurements. To minimize the influence of the paper on the durability of prints, we chose a permanent paper. For the latter, we also measured its basic properties and monitored the effect of light on the paper itself.

## 2. Results and Discussion

### 2.1. Chromatography

**Figure 1:**
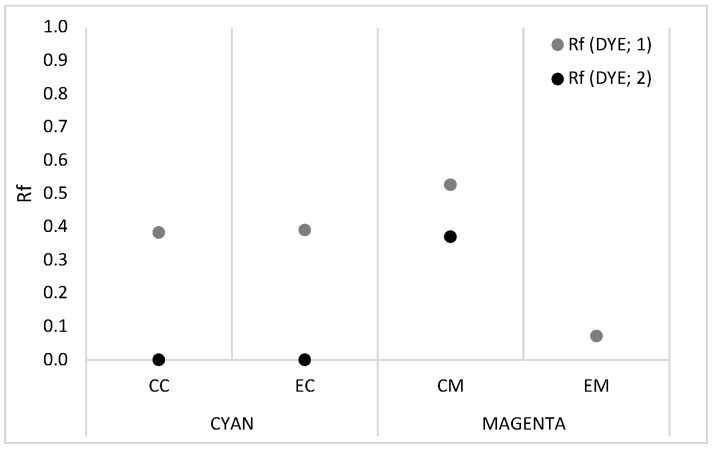
TLC analysis of cyan (CC and EC) and magenta (CM and EM) inks.

According to TLC analysis (Figure 1), the inks for inkjet printing consist of several different color components, but only one of the magenta inks (EM) included in our research contains a single colorant.

With GC/MS, we detected the presence of additives that are used to optimize the ink’s properties. In the case of inks included in printer T1 (CC and MC), glycerine, divinyl sulfone, 1,5-pentadiol, 2-pyrrolidone, and ethanol were detected; however, ink samples of the second printer T2 (EC and EM), in addition to glycerine, also contain trimethylene glycol and ethanol. Inks often contain different solvents, affecting surface tension, ink drying, and other properties [29]. By adding glycerine, the manufacturers control the ink’s viscosity and drying time of the prints [30,31]. The addition of 2-pyrrolidone prevents water evaporation and sedimentation of colored components in ink [31]. 1,5-pentadiol is used to prevent print wrinkling [31]. The presence of sulfone group components indicates the presence of surfactants in ink [32].

### 2.2. Photodegradation of Ink in Aqueous Solution

According to TLC analysis, both cyan inks are composed of a mixture of at least two color components. The most commonly used cyan dye for inkjet printing is a direct dye with a phthalocyanine structure, C.I. DB199, and an absorption maximum at 610 (594) nm, which has good light fastness [15,23,32]. According to the results, the absorption spectra suggest the presence of a direct dye with a phthalocyanine structure, C.I. DB86, which is characterized by absorption in the ranges of 660–664 and 620–624 nm and, like C.I. DB199, has excellent light fastness [15].

**Figure 2:**
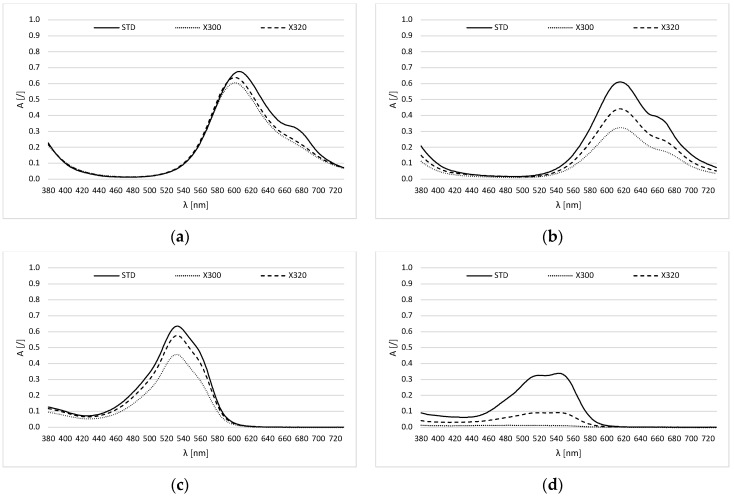
Absorption spectra of ink solutions ((**a**) CC; (**b**) EC; (**c**) CM; (**d**) EM) before (–––––) and after 144 h of exposure with X300 (∙∙∙∙∙) and X320 (- - - - -).

For the CC ink solution, slightly lower absorption peaks were observed after illumination using X300 and X320 (Figure 2a). The stability of the CC ink in the solution is excellent. A few color shifts were detected in the spectrum. Nevertheless, it can be pointed out that the peak at 606 nm has shifted toward shorter wavelengths.

Given that both cyan samples presumably use phthalocyanine colorants, the fastness of the EC ink is surprisingly poor (Figure 2b). In particular, the negative effect of near-UV radiation is evident, as the maximum absorption values were reduced by almost 50% of the initial value.

Figure 2c,d show that the two magenta inks have entirely different compositions. According to the TLC analysis, CM ink can be expected to contain at least two colorants, whereas EM ink contains only one.

The comparison of absorption maxima shows that CM ink very likely contains the reactive red dye C.I. RR120, with a characteristic absorption maximum at 530 nm, and/or the basic violet dye C.I. BV10, with an absorption maximum at 543 nm [33]. C.I. RR120 has a diazo structure and good lightfastness, whereas C.I. BV10 has a xanthene structure and very poor lightfastness [34]. Thus, combinations of colorants with different structures and different fastness properties are often seen. Evidently, by mixing them, properties of one colorant affect the properties of the other and thus often impact the overall fastness of the ink. Given the absorption spectrum, it can be assumed that the manufacturer of this ink had precisely this in mind, as the stability of CM ink in solution was surprisingly good.

The stability of EM ink in an aqueous solution (Figure 2d) was significantly weaker in comparison to CM ink. TLC analysis shows the presence of only one color component. Since the spectrum of EM ink is extremely broad with two absorption maxima at 519 and 544 nm, it can be assumed that the ink contains the reactive red dye C.I. RR180, which is characterized by two absorption maxima at 544 and 520 nm [35]. C.I. RR180 has an azo structure and is considered the most generally used red magenta dye, which has good lightfastness and is often used alone or in combination—usually with C.I. AR52 [36,37].

Table 1 shows the calculated ink amount (IA) (Equation (3)) and half-life values after 144 h of irradiation with a xenon lamp at the absorption maximum for each solution. From the calculated values, we can determine the negative effect on the stability of the dye and the aqueous solution, as well as the time required to reduce the initial amount of dye by half. As expected, the illumination with source X320 (λ > 320 nm) had less impact on the stability of the colorant in the solution. Despite the phthalocyanine structure of the cyan dye, it can be observed that cyan CC is significantly more stable than cyan EC. We expected lower stability for magenta, but magenta CM was relatively stable in an aqueous solution.

### 2.3. Photodegradation of Ink on the Substrate

**Figure 3:**
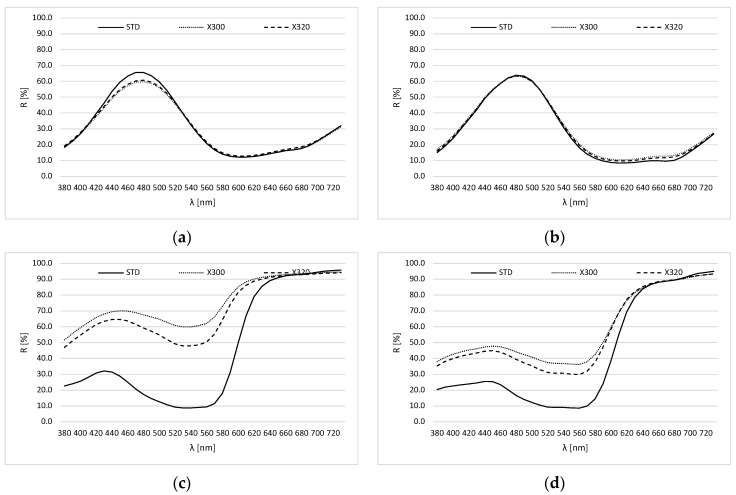
Reflection spectra of prints ((**a**) CC; (**b**) EC; (**c**) CM; (**d**) EM) before (–––––) and after 144 h of exposure to illumination with sources X300 (∙∙∙∙∙) and X320 (- - - - -).

From Figure 3, it can be observed that cyan ink was significantly more stable on the substrate than in an aqueous solution, as the change of spectra is less obvious.

Given that the changes on the prints, especially the magenta prints, were evident across the entire visible part of the spectrum, we decided to calculate the IA on the prints at the absorption maximum (Figure 3). Therefore, the calculations of IA and t_1/2_ on substrate are presented in Table 2. After 144 h of exposure with X300 and X320, we found that the cyan print remained well stable on the substrate, and the t_1/2_ of the cyan prints exceeded 1000 h of exposure. However, for magenta prints, a more significant effect of shortwave radiation (X300) on IA and t_1/2_ can be observed.

The color differences of the paper during the illumination were also monitored. These values are presented along with the different prints in Table 3. According to the CIE b^*^ value, the paper was slightly yellowish before exposure (Table 4). After 144 h of illumination with X300 and X320, some changes in the paper’s color can be observed; however, these changes are negligible and not visible to the naked eye (ΔE*_00_ < 0.5).

The cyan prints show relatively small color differences after 144 h of exposure.

Regardless of the wavelength range of the light source, it can be observed that CC print slightly darkens during the exposure. However, in the case of the EC print, we observed that the sample has become slightly redder compared to the standard (Δa* > 0).

In the case of magenta prints, significant color differences were observed. In general, the magenta samples became lighter and less red as value a* shifted towards green.

## 3. Materials and Methods

### 3.1. Inkjet Inks and Preparation of Samples

For the experimental work, we included two widely used printers and their corresponding inks from two manufacturers. The first printer was the Canon Pixma iP7250 (T1) (Canon Inc., Tokyo, Japan), and the second was the Epson L130 (T2) (Epson, Nagano, Japan). Both printers use dye-based inks for printing. The inks were extracted from the cartridges using a syringe and stored in tightly sealed glass vials. The inks were appropriately labelled as CC and CM for Canon, and EC and EM for Epson, where the first letter stands for printer manufacturer (C for Canon, E for Epson), and the second letter represents the color (C for cyan and M for magenta).

Before illumination, color samples were diluted 1:3000 with water. Prints were prepared using the Printer Driver RIP for Mac OS X (Apple, Cupertino, CA, USA) and printed on paper at a resolution of 2400 dpi.

### 3.2. Paper

In order to minimize the effects of the paper on the print, we selected a paper manufactured according to the standards for permanent paper (EN ISO 9706 [38] and ISO 11108 [39]). The manufacturer of the selected permanent paper was the Pulp and Paper Institute in Ljubljana, Slovenia. Some essential properties are shown in Table 4.

### 3.3. Chromatography (TLC and GC/MS)

For thin-layer chromatography (TLC), aluminum plates coated with silica gel and a fluorescent indicator (Sigma-Aldrich, Merck, Burlington, MA, USA) were used. Due to inadequate results in the mobile phase, we selected two solvent mixtures. Therefore, a mixture of ethyl methyl ketone, acetone, and water (7:5:3 *v*/*v*) was used for the cyan sample, while a combination of ethyl acetate, ethanol, and water (70:35:30 *v*/*v*) was used for the magenta sample. Based on the chromatograms, the retention factor (*R_f_*) was calculated using Equation (1):

(1) $$R_{f}=d_{s}/d_{mf},$$

where *d_s_* represents the distance travelled by the solute and *d_mf_* is the distance travelled by the solvent.

For gas chromatography (GC), an Agilent HP 6890 instrument (HP, Palo Alto, CA, USA) with a mass selective detector (GC/MS) and helium as carrier gas was used. The GC/MS analysis was carried out under the following conditions: a preheated oven (80 °C) and an injector system at 250 °C. The oven temperature program was pre-set to heat at a rate of 20 °C/minute up to 270 °C, after which it was maintained at a constant temperature. For injection, a 2 μL solution of ink in methanol was prepared.

### 3.4. Lightfastness of Colour Samples

To determine the lightfastness of color samples, a Xenotest Alpha (Atlas, Mount Prospect, IL, USA) with a xenon arc lamp was used along with two filters: Xenochrome 300 (X300) and Xenochrome 320 (X320), at a constant temperature of 35 °C and RH of 35%. With the X300 filter, shorter wavelengths were included (λ > 300 nm), and the spectrum simulated daylight in open spaces. The second filter, X320, included wavelengths above 320 nm and simulated light behind window glass. Samples were exposed to xenon light for 144 h.

**Figure 4:**
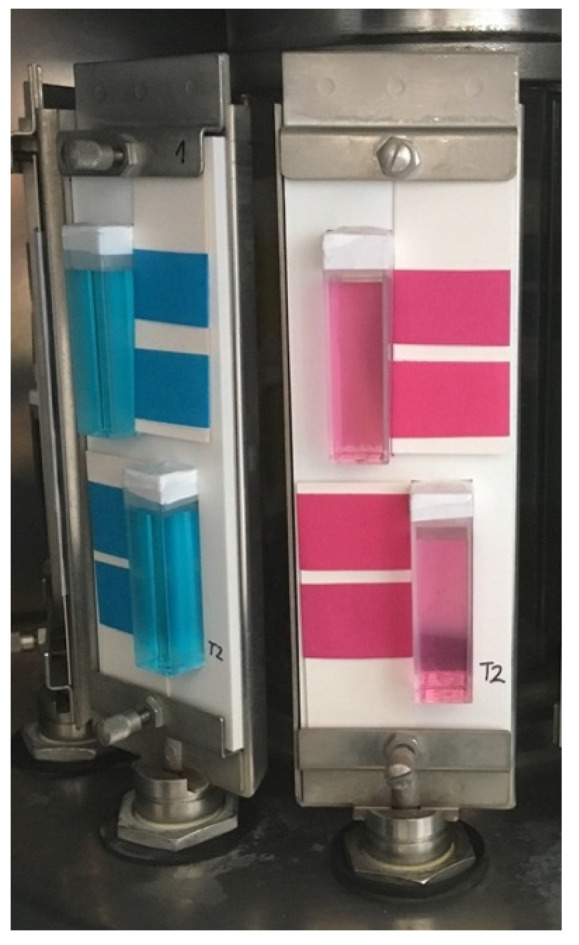
The arrangement of the printed and aqueous samples on the carrier in the Xenotest Alpha (Atlas, USA).

Using a Forex plate, we made special carriers to illuminate solutions and prints under the same conditions. Figure 4 shows the arrangement of the samples in the Xenotest Alpha (Atlas, USA).

### 3.5. Spectrophotometrical Measurements

The influence of light on the fastness of color samples was studied spectrophotometrically. Measurements of aqueous solutions were performed using a UV/VIS spectrophotometer Cary 1E (Varian, Palo Alto, CA, USA). For measuring aqueous solutions, we used UV cuvettes (Ratiolab, Dreieich, Germany). Measurements of transmission were performed in two repetitions before and after 144 h of illumination in the range of 200 to 800 nm with an increment of 1 nm.

Measurements of prints were performed using the i1 PRO spectrophotometer (X-rite, Grand Rapids, MI, USA) according to standard ISO 13655 [40], with 45°a:0° measuring geometry, white backing, D65 illuminant, and a 10° standard observer. The results represent the average values of three measurements. The color differences which appeared on the prints after exposure were calculated using the CIEDE2000 Equation (2) [41].

(2) $${∆E}_{00}^{*}=\sqrt{{\left(\frac{{∆L}^{\prime }}{k_{L}S_{L}}\right)}^{2}+{\left(\frac{{∆C}^{\prime }}{k_{C}S_{C}}\right)}^{2}+{\left(\frac{{∆H}^{\prime }}{k_{H}S_{H}}\right)}^{2}+R_{T}\left(\frac{{∆C}^{\prime }}{k_{C}S_{C}}\right)\left(\frac{{∆H}^{\prime }}{k_{H}S_{H}}\right)},$$

For all samples, the ink amount (*IA*) in solution and on prints was calculated using Equation (3), and the half-life (t_1/2_) was calculated after exposure. In an aqueous solution, it can be expected that the degradation reaction follows first- or pseudo-first-order kinetics, as the water concentration changes were negligibly small [10,19]. Thus, from the slope of the straight line, we have calculated the time required for dye concentration to decrease to half of its initial value—the half-life (t_1/2_) was estimated after 144 h of illumination.

(3) $$IA\left[\%\right]=A_{i}/A_{0}\times 100\%=\frac{log1/R_{i}}{log1/R_{0}}\times 100\%$$

## 4. Conclusions

According to the results, the degradation of dyes in solution depends on internal factors such as the chemical structure of the dye, the catalytic effect of dyes, and the presence of solvents in the carrier solution. In the case of prints, however, the influence of internal factors is altered. The co-substances of the ink are assumed to evaporate while the dye binds to the substrate surface, which also affects the degradation process indirectly. The results show that shortwave UV radiation did not significantly affect the cyan ink on substrate. However, a significant destructive effect of UV radiation was observed for EC cyan ink in the aqueous solutions. For magenta samples, the influence of shortwave UV radiation is evident both on the prints and in the aqueous solutions. The only exception was the magenta CM, which shows relatively good lightfastness in an aqueous solution.

Color metrics has proven to be an effective method for the characterization of photodegradation based on optical properties. By considering the combined effect of internal and external factors which affect the complex process of photodegradation, the half-lives and degradation rates of dyes in solutions and prints can be determined. Based on the results, we can establish that the degradation of prints cannot be evaluated solely by testing the fastness of the colorants or their solutions. The paper as a substrate must be included in the analysis, as it significantly influences the photodegradation of the print.

Our research has practical implications for industries related to printed materials, such as packaging, reproduction, and document preservation. According to our results, print substrates should be considered as active participants in degradation processes that affect the longevity of printed materials. This is especially relevant for conservation science and industries concerned with long-term color stability. Future research should focus on how different paper compositions (e.g., coated vs. uncoated, acidic vs. alkaline) affect dye degradation. Also, diverse real-world aging conditions should be considered to improve predictive models of print lightfastness.

## Figures and Tables

**Table 1:** Calculated ink amount (IA [%]) and half-life (t_1/2_ [h]) in aqueous solution at absorption maximum (λ_max_ [nm]) after 144 h of illumination with sources X300 and X320.

Sample Name	λ_max_ [nm]	X300		X320	
		IA [%]	t_1/2_ [h]	IA [%]	t_1/2_ [h]
CC	610	87	693	91	1155
EC	641	53	144	72	301
CM	533	72	301	91	866
EM	544	3	28	28	75

**Table 2:** Calculated ink amount (IA [%]) and half-life (t_1/2_ [h]) on substrate at absorption maximum (λ_max_ [nm]) after 144 h of illumination with sources X300 and X320.

Sample Name	λ_max_ [nm]	X300		X320	
		IA [%]	t_1/2_ [h]	IA [%]	t_1/2_ [h]
CC	610	100	1155	97	1386
EC	640	91	1155	94	1733
CM	530	21	49	30	60
EM	540	42	80	49	99

**Table 3:** Color differences on paper and prints after 144 h of illumination with sources X300 and X320.

	X300					X320				
	Paper	CC	EC	CM	EM	paper	CC	EC	CM	EM
ΔL*	0.09	−0.89	1.28	31.31	20.67	0.05	−0.21	0.80	26.83	17.53
Δa*	0.16	−0.15	2.65	−44.02	−30.09	0.19	0.37	1.57	−39.90	−24.01
Δb*	−0.53	3.66	1.67	1.19	−0.14	−0.68	3.80	1.22	−2.77	−1.32
ΔE*_00_	0.37	1.47	1.48	30.46	21.34	0.46	1.25	0.92	25.95	17.85

**Table 4:** Permanent paper properties.

Paper Characteristics	Paper
Grammage [g/m^2^]	80
Thickness [μm]	102
Ash content [%]	11.7
CIE whiteness	81.85
Yellowness	5.83
CIE L*	97.79
CIE a*	−0.19
CIE b*	2.83
Roughness	3.06

## Data Availability

The raw data supporting the conclusions of this article will be made available by the authors on request.
